# Sequence diversity and differential expression of major phenylpropanoid-flavonoid biosynthetic genes among three mango varieties

**DOI:** 10.1186/s12864-015-1784-x

**Published:** 2015-07-30

**Authors:** Van L. T. Hoang, David J. Innes, P. Nicholas Shaw, Gregory R. Monteith, Michael J. Gidley, Ralf G. Dietzgen

**Affiliations:** School of Pharmacy, The University of Queensland, Brisbane, Queensland Australia; Centre for Nutrition and Food Sciences, Queensland Alliance for Agriculture and Food Innovation, The University of Queensland, Brisbane, Queensland Australia; Department of Agriculture and Fisheries, Agri-Science Queensland, Brisbane, Queensland Australia

**Keywords:** Mango fruit, Expressed sequence tags, Phenylpropanoid-flavonoid pathway, Nucleotide diversity, Gene expression

## Abstract

**Background:**

Mango fruits contain a broad spectrum of phenolic compounds which impart potential health benefits; their biosynthesis is catalysed by enzymes in the phenylpropanoid-flavonoid (PF) pathway. The aim of this study was to reveal the variability in genes involved in the PF pathway in three different mango varieties *Mangifera indica* L., a member of the family Anacardiaceae: Kensington Pride (KP), Irwin (IW) and Nam Doc Mai (NDM) and to determine associations with gene expression and mango flavonoid profiles.

**Results:**

A close evolutionary relationship between mango genes and those from the woody species poplar of the Salicaceae family (*Populus trichocarpa*) and grape of the Vitaceae family (*Vitis vinifera*), was revealed through phylogenetic analysis of PF pathway genes. We discovered 145 SNPs in total within coding sequences with an average frequency of one SNP every 316 bp. Variety IW had the highest SNP frequency (one SNP every 258 bp) while KP and NDM had similar frequencies (one SNP every 369 bp and 360 bp, respectively). The position in the PF pathway appeared to influence the extent of genetic diversity of the encoded enzymes. The entry point enzymes phenylalanine lyase (PAL), cinnamate 4-mono-oxygenase (C4H) and chalcone synthase (CHS) had low levels of SNP diversity in their coding sequences, whereas anthocyanidin reductase (ANR) showed the highest SNP frequency followed by flavonoid 3’-hydroxylase (F3’H). Quantitative PCR revealed characteristic patterns of gene expression that differed between mango peel and flesh, and between varieties.

**Conclusions:**

The combination of mango expressed sequence tags and availability of well-established reference PF biosynthetic genes from other plant species allowed the identification of coding sequences of genes that may lead to the formation of important flavonoid compounds in mango fruits and facilitated characterisation of single nucleotide polymorphisms between varieties. We discovered an association between the extent of sequence variation and position in the pathway for up-stream genes. The high expression of PAL, C4H and CHS genes in mango peel compared to flesh is associated with high amounts of total phenolic contents in peels, which suggest that these genes have an influence on total flavonoid levels in mango fruit peel and flesh. In addition, the particularly high expression levels of ANR in KP and NDM peels compared to IW peel and the significant accumulation of its product epicatechin gallate (ECG) in those extracts reflects the rate-limiting role of ANR on ECG biosynthesis in mango.

**Electronic supplementary material:**

The online version of this article (doi:10.1186/s12864-015-1784-x) contains supplementary material, which is available to authorized users.

## Background

The PF pathway in fruits and vegetables is of great interest because it leads to compounds that exhibit important health benefits, including antioxidant, antibacterial, anti-inflammatory and anticarcinogenic effects [1]. This pathway is highly conserved among diverse plant species and is well defined in *Arabidopsis thaliana* (Family: Brassicaceae) and several temperate fruit species including grape, apple, berry and olive fruits [2–8]. Mango is the fifth largest fruit crop in the world [9] and a good candidate for research on phytochemical improvement to create better fruit value, because of its high genetic diversity [10] and the current lack of breeding for nutritional quality. Mango fruits are rich sources of phytochemicals, including antioxidants and other potential health-promoting compounds [11]. A comparative bioactivity study between the three genetically diverse mango varieties KP, IW and NDM showed differential effects on lipid accumulation in 3 T3-L1 pre-adipocyte mouse embryo fibroblasts [12]. A parallel study of mango chemical profiles in the same extracts has shown a clear difference in major phenolic components between these varieties [13]. The total phenolic content of Kensington Pride, Irwin and Nam Doc Mai peel and flesh extracts has been recently reported with Nam Doc Mai peel extract containing the highest amount of polyphenolics [12].

Mango fruit studies have previously largely focused on the ripening process [14–16], volatile composition [17, 18], postharvest treatment and fruit quality [19] at the physiological level. Earlier studies have isolated 18 genes associated with the physiology and biochemistry of mango fruits and their gene expression was profiled during fruit development [20]; no detailed sequence information was reported. Recently, the first mango genome and transcriptome data were reported [21–23]. More than 13,500 unigenes of mango were assigned to 293 KEGG pathways [22] however genes were limited to the chloroplast genome and expression in leaf tissues. In another study, transcriptome sequencing of a mixed fruit sample containing flesh and peel of mango variety Zill during fruit ripening was reported [21]. These authors assembled 54,000 transcripts and matched mango transcripts to 2754 proteins [20], but did not correlate expressed mango genes and mango phytochemicals. A mango fruit peel transcriptome in response to hot water treatment, which included flavonoid genes has also been reported [23].

The level of nucleotide sequence variation, the position of the gene products in the pathway and the expression levels of genes all contribute to the abundance and chemical diversity of bioactive compounds [24]. The most frequent type of sequence variations are single nucleotide differences, which are generally referred to as single nucleotide polymorphisms (SNPs) [25]. SNPs within a coding sequence may change amino acid sequence and therefore potentially affect protein function [26, 27]. Sequence diversity of enzymes in the phenylpropanoid-flavonoid (PF) pathway has been studied in detail in the model species *Arabidopsis thaliana* [6, 28] and in important field crops such as maize (*Zea mays*) (Family: Poaceae) [29] and barley (*Hordeum vulgare*) (Family: Poaceae*)* [30].

**Fig. 1:**
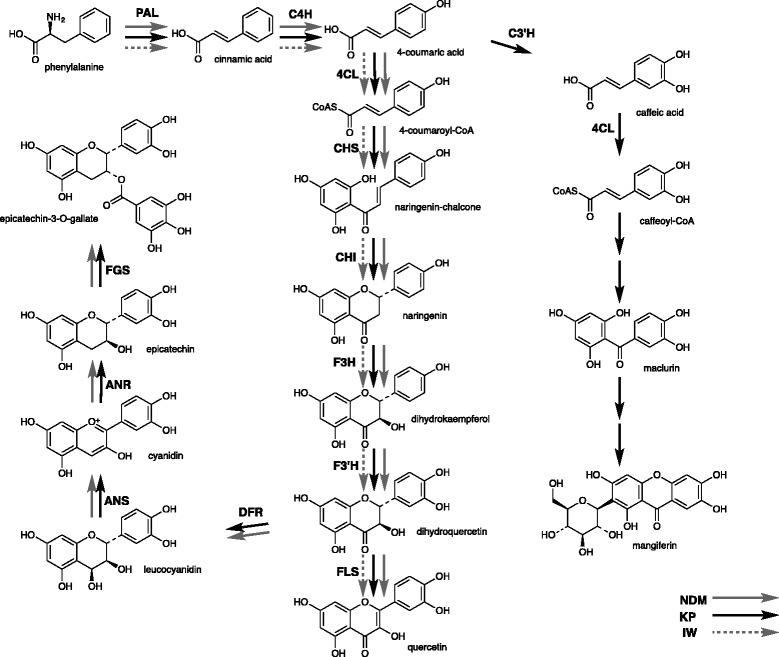
Biosynthesis pathway leading to major flavonoid compounds in mangoes. The black, grey and dash grey arrows indicate major flavonoid compounds and their pathway enzymes as identified in KP, NDM and IW fruits, respectively. PAL: phenylalanine ammonia lyase; C4H: cinnamate 4-mono-oxygenase; 4CL: 4-coumarate: CoA ligase; C3’H: *p*-coumaroyl ester 3′-hydroxylase; CHS: chalcone synthases; CHI: chalcone isomerase; F3H: flavanone 3-hydroxylase; F3’H: flavonoid 3’-hydroxylase; DFR: dihydroflavonol 4-reductases; FLS: flavone synthase; ANS: anthocyanidin synthase; ANR: anthocyanidin reductase; FGS: flavan-3-ol-gallate synthase

In this paper, we report the sequence variability of genes involved in the PF biosynthesis of mangoes of different genetic origin and the relative levels of gene expression in order to assess the potential role of these genes in governing accumulation of flavonoid compounds in mango fruits. Our study was aimed at investigating health benefits of consuming mango fruit; for this reason, we studied the expression of mango genes and associated bioactive polyphenolic compounds at the mature ripe fruit stage. Previously, the Mango Genomics Initiative of the Queensland Department of Agriculture and Fisheries generated ~25,000 expressed sequence tags (ESTs) from cDNA libraries of KP root, leaf, flower and fruit tissues as well as from IW leaf tissue [31] which enabled this comparative study. Based on a combination of results from automated data extraction from the Kyoto Encyclopedia of Genes and Genomes (KEGG; www.genome.jp/kegg/) and the presence of selected flavonoids in our mango extracts [13], we also propose here a mango fruit PF pathway, with particular emphasis on the mango signature compounds mangiferin and epicatechin-3-*O*-gallate [32, 33] (Fig. 1).

## Results and discussion

### Identification of PF pathway candidate genes from a mango EST library

To identify PF biosynthesis genes, we performed an *in silico* screen of the mango EST database sequences. A total of 12 candidate genes for PAL, C4H, *p*-coumaroyl ester 3′-hydroxylase (C3’H), 4-coumarate:CoA ligase (4CL), CHS1, CHS2, flavanone 3-hydroxylase (F3H1, F3H2), F3’H, dihydroflavonol 4-reductases (DFR), anthocyanidin synthase (ANS) and ANR with annotations matching enzymes in the PF pathway leading to ECG and mangiferin biosynthesis were identified. This information was used to design PCR strategies for the amplification of the coding regions of these genes using cDNA prepared from KP, IW and NDM fruit. All individual sequences have been deposited in GenBank (Table 1).

**Table 1:** List of Genbank accession numbers of mango genes

Gene	Mango variety	Accession numbers
PAL	KP	KF956008
	IW	KF956009
	NDM	KF956010
C4H	KP	KF956011
	IW	KF956012
	NDM	KF956013
4CL	KP	KF956014
	IW	KF956015
	NDM	KF956016
C3’H	KP	KF956017
	IW	KF956018
	NDM	KF956019
CHS1	KP	KF956020
	IW	KF956021
	NDM	KF956022
CHS2	KP	KF956023
	IW	KF956024
	NDM	KF956025
F3H1	KP	KF956026
	IW	KF956027
	NDM	KF956028
F3H2	KP	KF956029
	IW	KF956030
	NDM	KF956031
F3’H	KP	KF956032
	IW	KF956033
	NDM	KF956034
DFR	KP	KF956035
	IW	KF956036
	NDM	KF956037
ANS	KP	KF956038
	IW	KF956039
	NDM	KF956040
ANR	KP	KF956041
	IW	KF956042
	NDM	KF956043

**Fig. 2:**
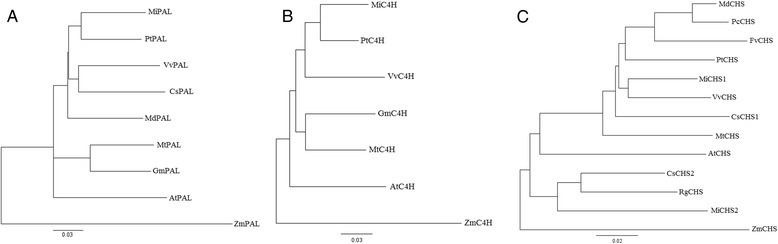
Neighbour-joining trees of selected plant PAL (**a**), C4H (**b**) and CHS (**c**) proteins. Bootstrap values above 50 % are indicated at nodes. The tree is drawn to scale, with branch lengths in the same units as those of the evolutionary distances that were used to infer the phylogenetic tree. PtPAL (*Populus trichocarpa* GenBank: XP_002315308), VvPAL (*Vitis vinifera* GenBank: XP_002272926), CsPAL (*Citrus sinensis* GenBank: XP_006488063), MdPAL (*Malus domestica,* Family: Rosaceae, GenBank: AFG30054), MtPAL (*Medicago truncatula,* Family: Fabaceae, GenBank:P_003590471), GmPAL (*Glycine max* GenBank: NP_001236956), AtPAL (*Arabidopsis thaliana* GenBank:NP_187645), ZmPAL (*Zea mays* GenBank: NP_001105334), PtC4H (*Populus trichocarpa* GenBank:XP_002319974), VvC4H (*Vitis vinifera* GenBank: XP_002266238), MtC4H (*Medicago truncatula* GenBank: XP_003616037), GmC4H (*Glycine max* GenBank: NP_001237317), AtC4H (*Arabidopsis thaliana* GenBank: NP_180607), ZmC4H (*Zea mays* GenBank: NP_001149158), MdCHS (*Malus domestica* GenBank: ACJ54531), FvCHS (*Fragaria vesca*, Family: Rosaceae, GenBank: XP_004306542), PcCHS (*Pyrus communis*, Family: Rosaceae, GenBank: AAX16494), PtCHS (*Populus trichocarpa* GenBank: XP_002303821), VvCHS (*Vitis vinifera* GenBank: AEP17003), CsCHS1 (*Citrus sinensis* GenBank: Q9XJ57), CsCHS2 (*Citrus sinensis* GenBank: Q9XJ58), MtCHS (*Medicago truncatula* GenBank: XP_003601647), AtCHS (*Arabidopsis thaliana* GenBank: P13114), RgCHS (*Ruta graveolens*, Family: Rutaceae, GenBank:Q9FSB9), ZmCHS (*Zea mays* GenBank:NP_001149022)

Phylogenetic comparisons of the translated consensus sequences of mango PF pathway proteins against corresponding sequences of a selected plant reference protein set, available through GenBank, revealed that the two closest neighbours of mango were *P. trichocarpa* and *V. vinifera* (Fig. 2; data not shown). Similar results were reported recently based on the evolutionary relationship between actins from those plant species [34] and on mango chloroplast genes which showed a close relationship between mango, *P. trichocarpa*, *V. vinifera* and *Citrus sinensis* (Family: Rutaceae) as closest neighbour [22].

### SNPs analysis

Multiple sequence alignments showed that the PF pathway genes of the three mango varieties tested have similar lengths, and high sequence identity with a range of 95.7–99.9 %, implying conservation of each gene sequence between varieties. There were no deletions or insertions in any of the amplified gene fragments and no mutations that introduced premature stop codon that would generate a truncated protein. However, there was variation in sequences between the three mango varieties studied. Overall, we detected 145 polymorphic sites across the entire 46,560 bp of nucleotide sequence of the 12 PF biosynthetic genes analysed. Of these, 84 were synonymous SNPs or silent changes and 61 were non-synonymous SNPs, which would lead to changes in the encoded amino acid. The IW variety had approximately 30 % more SNPs than the other two varieties although IW and KP had the same number of non-synonymous SNPs. The overall frequency of SNPs in the three mango varieties tested was one in every 316 bp. Variety IW had the highest SNP frequency (one SNP every 258 bp) while KP and NDM had similar frequencies (one SNP every 369 bp and 360 bp, respectively). This SNP frequency is close to that reported in coding regions in black cottonwood (*P. trichocarpa*) (one SNP per 229 bp) [35], higher than in soybean (*Glycine max*) (Family: Fabaceae) (one SNP every 504 – 609 bp) [36] but lower than highly polymorphic plant species such as maize (*Zea mays*) or grape (*V. vinifera*) (approximately one SNP every 69 bp) [37, 38].

Table 2 summarises the sequenced length of each gene fragment, the number of SNPs, the number of non-synonymous SNPs and the number of SNPs per 1000 bp of individual genes in each mango variety. Overall, levels of polymorphism varied across genes within varieties and between varieties; and variation was lower at non-synonymous sites than at synonymous sites between varieties. Genes of the variety IW had the highest levels of SNP diversity, especially in the 4CL gene (11.6 SNPs in 1000 bp). This variety has a different phytochemical content compared to KP and NDM [13]. The gene with the lowest number of SNPs per 1000 bp was F3H1 (0.8 SNPs in 1000 bp) while the gene with the highest SNP frequency was ANR (8.8 SNPs in 1000 bp), followed by F3’H (Table 2). The nucleotide and non-synonymous variation was found to be low in PAL, C4H and CHS genes (Table 2). These three genes are located at the entry to the PF biosynthetic pathway. A previous study by Lu and Rausher [39] that analysed anthocyanin pathways in monocot and dicot species also showed that downstream genes exhibited significantly greater divergence rates than upstream genes. Similar patterns of variation were found in genes of the carotenoid biosynthetic pathway for tomato, carrot and rice [40]. This can be explained because upstream enzymes provide precursors for various groups of end-products and control the flux into the pathways. Changes in their coding regions could have major effects on plant phytochemical biosynthesis and therefore could greatly affect the accumulation of various end products.

**Table 2:** Summary of alignment length, number of SNPs, number of non-synonymous SNPs and the frequency of SNPs per 1,000 bp of 12 genes of the phenylpropanoid-flavonoid pathways in each of three mango varieties

Gene	Length (bp)	KP			IW			NDM			SNPs per 1,000 bp
		No. of SNPs	No. of non-synonymous	SNPs per 1,000 bp	No. of SNPs	No. of non-synonymous	SNPs per 1,000 bp	No. of SNPs	No. of non-synonymous	SNPs per 1,000 bp	
PAL	2,123	2	1	0.9	5	3	2.4	4	1	1.9	1.9
C4H	1,499	0	0	-	0	0	-	6	5	4	1.3
4CL	1,637	1	1	0.6	19	9	11.6	0	0	-	4.1
C3’H	1,524	8	1	5.2	6	0	3.9	5	1	3.3	4.2
CHS1	1,171	3	2	2.5	6	0	5.1	1	0	0.9	2.8
CHS2	1,179	0	0	-	1	0	0.8	2	0	1.7	0.8
F3H1	1,082	0	0	-	1	1	0.9	0	0	-	0.3
F3H2	1,075	10	6	9.3	6	4	5.6	2	0	1.9	5.6
F3’H	1,548	9	5	5.8	7	2	4.5	12	6	7.7	6.0
DFR	1,002	1	1	1	0	0	0	1	0	1	0.7
ANS	919	1	0	1.1	4	2	4.4	1	0	1.1	2.2
ANR	761	6	5	7.9	5	1	6.5	9	4	11.8	8.8

The sequence positions of identified mutations and the alleles present in the three mango varieties are presented in Table 3. Most SNPs occurred outside of the reported conserved regions thought to be important for enzyme function. There has been no previous report about the effects of SNPs at these positions in other plant species. However, even SNPs that do not change the amino acid sequence or SNPs that do not alter protein activity, can still be useful as genetic markers [41]. Additionally, most of the genes showed the presence of one or two allelic sequences, which confirms the heterozygosity of the KP, IW and NDM genomes [31].

**Table 3:** Characteristics of non-synonymous SNPs of 12 genes of the phenylpropanoid-flavonoid pathways in three mango varieties

Gene	SNP position	Context	Amino acid replacement	KP	IW	NDM
PAL	399	(A/G)TG	Ser – Gly	A	G	A
	702	(A/G)GT	Ser – Gly	A	A	G
	718	G(A/T)G	Glu - Val	A/T	A	A
	1,387	C(T/C)T	Leu – Pro	T	T/C	T
	1,980	(G/A)CA	Ala – Thr	G	A	G
C4H	52	(G/A)TT	Val – Ile	G	G	G/A
	124	(C/G)TT	Leu – Val	C	C	C/G
	183	TT(A/C)	Leu – Phe	A	A	A/C
	205	(C/T)TT	Leu - Phe	C	C	C/T
	751	(C/A)AA	Gln – Lys	C	C	C/A
4CL	56	C(C/T)T	Pro – Leu	C/T	C	C
	206	G(T/C)C	Val – Ala	T	C	T
	247	(C/A)TG	Leu – Met	C	A	C
	304, 306	(C/G)G(C/T)	Arg - Gly	CGC	GTG	CGC
	433	(A/G)AA	Lys – Glu	A	G	A
	1,159	(C/G)CA	Pro – Ala	C	G	C
	1,290	GA(A/T)	Glu – Asp	A	T	A
	1,557	GA(T/A)	Asp – Glu	T	A	T
C3’H	797	G(T/C)C	Val – Ala	T	T	C
	803	CA(A/T)	Gln – His	T	A	A
CHS1	247	(G/A)TT	Val – Ile	A	G	G
	253	(G/C)AA	Glu – Gln	C	G	G
F3H1	902	T(A/G)C	Tyr – Cys	A	G	A
F3H2	14	A(C/G)T	Thr – Ser	C	G	C
	161	G(G/A)T	Gly – Asp	G	A	G
	271	(A/G)AA	Lys – Glu	G	A	A
	284	T(T/C)G	Leu – Ser	C	T	T
	509	A(A/T)G	Lys – Met	A	T	A
	626	GA(A/C)	Glu – Asp	C	A	A
	690	CA(G/C)	Gln – His	C	G	G
	796	(A/T)GC	Ser – Cys	T	A	A
	1,018	(G/T)CC	Ala – Ser	T	G	G
	1,067	C(A/G)G	Gln – Arg	A	G	A
F3’H	35	G(C/G)C	Ala – Gly	G	C	C/G
	61	(T/A)TA	Leu – Ile	A	T	A/T
	92	C(C/G)(T/C)	Pro – Arg	CGT	CCT	C(G/C)(C/T)
	115	(T/C)GG	Trp – Arg	C	T	C/T
	790	(C/A)(G/T)T	Agr – Ile - Ser	ATT	CGT	A(G/T)T
	863	G(G/A)A	Agr – Glu	G	G	A/G
	1,243	(C/A)TA	Leu - Ile	A	C	C
DFR	901	(A/G)AG	Lys – Glu	G	A	A
ANS	169	GA(G/C)	Glu – Asp	G	C	G
	633	G(G/A)G	Arg – Glu	G	A	G
ANR	49	(A/G)CC	Ala – Thr	A/G	A	A
	68	T(T/G)G	Leu – Trp	G/T	T	T
	128	G(A/G)T	Asp – Arg	A	A	G
	136	(C/A)AA	Gln – Lys	C	C	A
	172	(G/T)GT	Cys – Arg	T/G	G	G
	226	(C/T)CT	Cys – Pro	T/C	C	C
	329	G(A/G)G	Glu – Agr	A	A	G
	506	G(T/C)C	Val – Ala	T	T	C
	552	GA(G/T)	Glu - Asp	G	G	T
	653	T(T/A)A	Leu – Ser	T	T/C	C

### Transcriptional profiles of genes involved in biosynthesis of phenolic compounds in mango fruit

We used quantitative, reverse-transcription PCR (RT-PCR) to assess potential correlations between the expression of PF genes and the differences in chemical profiles of KP, IW and NDM. The expression patterns of the phenylpropanoid biosynthetic genes (PAL, C4H) and genes involved in the biosynthesis of various flavonoids (4CL, C3’H, CHS1, CHS2, F3H1, F3H2, F3’H, DFR, ANS and ANR) were examined in mango peel and flesh of ripe fruits with actin as an endogenous reference gene.

**Fig. 3:**
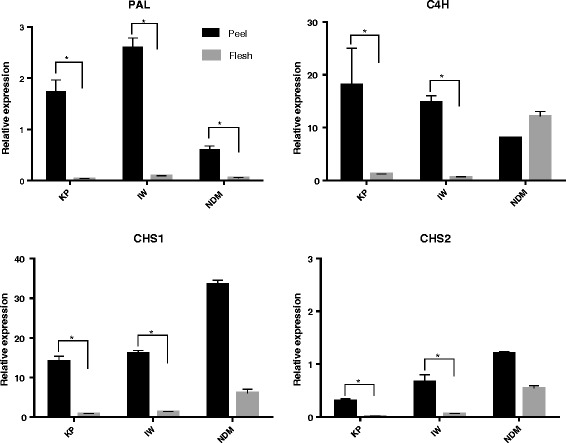
Relative expression levels of PAL, C4H and CHSs mRNAs in mango peel (black) and flesh (grey) determined by Real-time RT-PCR. For all panels the data represent mean ± SEM relative to the Actin control (*n* = 3, *P < 0.01)

Transcript levels of PF upstream genes differed significantly between mango peel and flesh. The expression of PAL gene in KP peel was approximately 45-fold higher than in the respective KP flesh, 30-fold higher in IW peel than flesh, and 12-fold higher in NDM peel than flesh, when expression was normalised to actin gene expression (Fig. 3). The expression of C4H and CHS genes in KP and IW peels were significantly (*P* < 0.01) higher than in flesh when expression was normalized to actin housekeeping gene (Fig. 3). There was no significant difference in the expression of C4H and CHS genes between NDM peel and flesh, indicating significant differences between varieties. PAL mediates carbon flux into the phenylpropanoid pathway to produce cinnamic acid, the substrate for the next step mediated by C4H (Fig. 1). These steps represent the connection between primary and secondary metabolism while the first committed step of the flavonoid biosynthesis pathway is mediated by CHS. Our previous studies have demonstrated that soluble polyphenolic contents expressed as gallic acid equivalents (GAE) in mango peel extracts varied between 634 and 868 mg (GAE) per g dry weight, and were higher than flesh extracts (181– 304 mg GAE per g dry weight) [33]. This study provides evidence of a coordinated up-regulation of up-stream biosynthesis pathway genes leading to high accumulation of phenolic compounds in mango peel. Similar patterns have been reported in tomato, where the expression of the upstream flavonoid biosynthesis genes including PAL and CHS exhibited significant correlation with accumulation of flavonoid compounds in peel; and only low levels of flavonoid-related transcripts were detected in the flesh [42, 43].

**Fig. 4:**
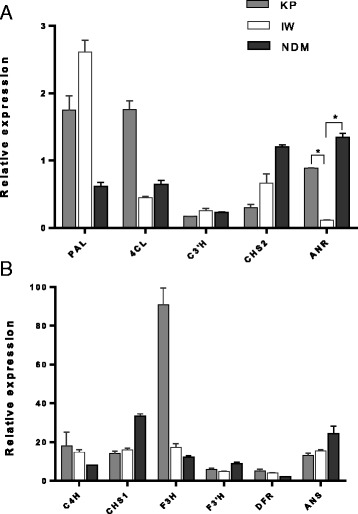
Relative expression levels of flavonoid biosynthetic genes in the peel of three mango fruit varieties determined by Real-time RT-PCR. **a** PAL, 4CL, C3’H, CHS1 and ANR. **b** C4H, CHS1, F3H, F3’H, DFR and ANS. For all panels the data represent mean ± SEM relative to the Actin control, (*n* = 3, *P < 0.01)

**Fig. 5:**
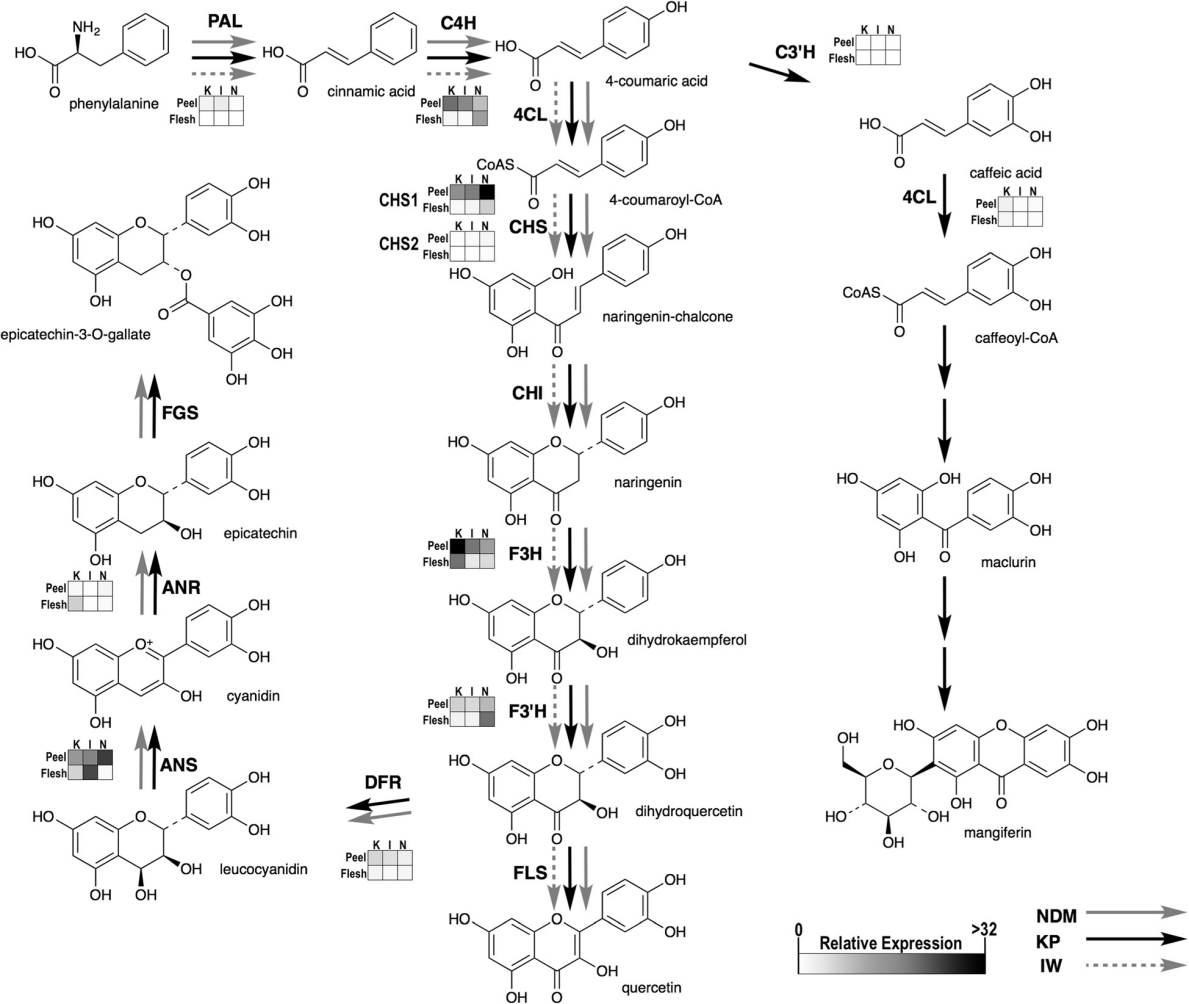
MapMan visualization of changes in expression of genes involved in the phenylpropanoid flavonoid metabolism in peel and flesh tissues of KP, NDM and IW fruits

Previous studies of gene expression in temperate fruits reported two peaks of flavonoid activity during fruit ripening, one in green fruit and the other in nearly ripe fruit [44–46]. In our mango study, the expression of 12 PF biosynthetic genes was detected in all mango peels at the ripe stage. Remarkably, a significant difference in transcript levels between varieties was observed for ANR (Fig. 4). Roles of CHS, F3H, DFR and ANS in the anthocyanin biosynthetic pathway have been reported in fruit plants such as olive, cacao and grape [7, 47, 48]. Additionally, some studies in green tea [49] and temperate fruits including apple, grape, and strawberry implied important roles of ANR in PA biosynthesis [50, 51]. In grape, the expression of ANS and ANR were co-regulated which showed their contribution to PA synthesis in fruit [52]. The over-expression of ANR in the Arabidopsis *banyuls* (*anr*) mutant restored PA synthesis in seeds [53]. During strawberry fruit development, the expression pattern of ANR was correlated with epicatechin production [50]. However, the expression pattern for ANR differed from plant tissues and fruit development [52, 54]. In this study, the ANR transcript level in IW was 8-fold lower than in KP and 12-fold lower than in NDM. There was no significant different in the expression of ANS in three mango varieties. A qualitative biochemical analysis among the three mango varieties demonstrated the presence of ECG in KP and NDM peel extracts but not in IW peel extract [13]. ANR is ultimately responsible for the biosynthesis of epicatechin and related compounds such as ECG (Fig. 1). Taken together, these results suggest an association between the higher expression of ANR genes in KP and NDM with the accumulation of ECG in extracts of these two mango varieties. It should be noted however that our study only analysed ripe stage fruits. The expression of genes involved in the phenylpropanoid flavonoid metabolism in peel and flesh tissues of KP, NDM and IW fruits were summarised and visualised by MapMan (Fig. 5).

## Conclusions

Our study shows that it is possible to discover candidate genes for many mango PF biosynthetic pathway enzymes through use of a mango fruit EST library. Phylogenetic analysis of pathway genes revealed a close evolutionary relationship between mango, *P. trichocarpa* and *V. vinifera*. This study has determined, for the first time, the SNP frequency in coding sequences of PF biosynthetic genes of KP, IW and NDM mangoes. This study established a positive relationship between total phenolic content and expression levels of PAL, C4H and CHS in mango fruit. The high expression of anthocyanin-related gene, ANR in KP and NDM peel compared to IW peel may explain the presence of ECG in KP and NDM peels, but its absence in IW peel. Further study could examine the possible association of the identified SNPs with expression differences. This combined knowledge will facilitate future breeding and selection of new mango varieties possessing tailored bioactive compound profiles with properties beneficial to health.

## Methods

### Preparation of mango fruit

KP, IW and NDM were provided by Dr Ian Bally from the Department of Agriculture and Fisheries' Southedge Research Station, Queensland, Australia and left to rest at 20 °C until they reached consumption ripeness as assessed by the sprung test [55] with some modification [12]. Fruits of each variety were collected from the same tree. The ripe fruits were cleaned with water and the seeds removed. The peel and flesh were then stored at −80 °C until total RNA was extracted.

### RNA extraction and cDNA synthesis

Total RNA was extracted using the cetyltrimethyl ammonium bromide (CTAB) method described by Chang et al. [56] with some minor modifications. Spermidine was omitted and only 1 % 2-mercaptoethanol was used. All solutions used in RNA extractions were treated with 0.1 % v/v diethyl pyrocarbonate (DEPC) and autoclaved to inactivate RNases. Ten mL of extraction buffer (2 % CTAB, 2.5 % polyvinylpolypyrrolidone, 2 M NaCl, 100 mM Tris–HCl pH 8.0, 25 mM EDTA pH 8.0 and 2 % of 2-mercaptoethanol added just before use) was heated at 65 °C in 50 mL tubes. Samples were extracted from three replicates, each replicate from three pooled mature fruits of each mango variety. The flesh or peel of pooled mango fruits (1 g), powdered in liquid nitrogen using a mortar and pestle, was added to the extraction buffer and the tube was incubated at 65 °C for 10 min. An equal volume of chloroform was added and the tube was inverted vigorously and centrifuged at 10,000 *g* for 10 min at 4 °C. Total RNA was precipitated by addition of 1 volume of 10 M LiCl to the supernatant. The mixture was incubated at 4 °C overnight and RNA was selectively pelleted by centrifugation at 10,000 *g* for 40 min at 4 °C. The pellet was resuspended in 5 mL of 70 % ethanol and the mixture was centrifuged at 10,000 *g* for 5 min at 4 °C, dried and resuspended in DEPC–treated water. RNA concentrations were determined using a NanoDrop® ND-1000 Spectrophotometer (Thermo Scientific), and the quality was confirmed on 1.2 % agarose gel using TAE buffer. RNA samples were treated with TURBO™ DNase (Ambion) in accordance with the manufacturer’s instruction and stored at −80 °C. About 1 μg of total RNA was reverse-transcribed by use of Superscript III First-strand Synthesis Mix (Invitrogen) with oligo (dT)_20_ primer in accordance with the manufacturer’s protocol.

### Gene discovery

Information on selected biosynthesis genes in mango (Fig. 1) was obtained from ESTs present in the Mango Genomics Database (http://mango.qfab.org), a proprietary database containing nucleotide sequence, phenotypic, and sensory information for mango. Sequence contig assemblies were used as initial source for analyses. Full-length EST sequences were obtained using M13 Forward (−20) and Reverse primers. Sanger sequencing was performed using Applied Biosystems 3730*xI* capillary sequencers by the Australian Genome Research Facility (AGRF, St Lucia, QLD, Australia). Nucleotide sequences from three colonies each were analysed using the Geneious v.5.1 (Biomatters, New Zealand) [57]. The amino acid sequences generated from the consensus sequences were compared using BLAST-P (http://blast.ncbi.nlm.nih.gov/Blast.cgi) [58] with default parameters against reference sequences available on the NCBI nucleotide databases (*e*-value ≤10^−5^) to confirm identities. All sequences were aligned using Geneious default values, with pair-wise parameters set at gap opening penalty 10, gap extension penalty 0.1 and multiple alignment parameters set at gap opening penalty of 10, gap extension 0.2. Sequences were deposited in GenBank with accession numbers shown in Table 1.

Primers were designed based on the open reading frames of all mango candidate genes identified from ESTs with default settings on the Primer feature in Geneious [59]. Primers were synthesised by Geneworks (Adelaide, Australia) (Additional file 1) and yielded expected amplicons with KP, IW and NDM cDNA. Fragments were amplified by PCR with Phusion HF (Finnzymes) using conditions recommended by the manufacturer, separated on 1.2 % agarose gels containing ethidium bromide with images captured on a Geldoc system (BioRad). PCR products were gel-purified using a Wizard® SV gel purification kit (Promega), ligated into PCR™4 Blunt-TOPO® vector (Invitrogen) and transformed into TOP10 competent cells (Invitrogen). Recombinant colonies were grown at 37 °C overnight on LB-Ampicillin (50 μg/mL) agar plates. Plasmid DNA was extracted from overnight liquid cultures using the GeneJET™ Plasmid Miniprep kit (Fermentas), quantified using a NanoDrop® and sequenced using M13 forward and reverse primers.

### Comparison of amino acid sequences and phylogenetic analyses

Amino acid sequences were aligned in Geneious using global alignment with default parameters [57]. Searches for homologous sequences were conducted using the BLAST-P module. To investigate the evolutionary relationships of candidate mango flavonoid pathway proteins to homologous proteins described from other plant species, phylogenetic trees were constructed using parsimony and/or genetic distance calculations Neighbour-joining and Bootstrap with 1000 replicates.

### Quantitative real-time PCR

Real-time RT-PCR was done in a RotorGene RG-6000 Thermal Cycler (Qiagen) to determine relative gene expression levels in peel and flesh of KP, IW and NDM fruits with actin (GenBank accession number HQ830244) as an endogenous reference gene [34, 60] with three independent biological replicates (three technical replicates for each sample) using Rotor-Gene SYBR Green PCR Kit (Qiagen). All primers were designed from available mango candidate gene sequences, with the Primer feature in Geneious [59] (S1) and verified by Netprimer software. Primers were synthesised by Geneworks. PCR conditions were 95 °C for 5 min followed by 40 cycles of 95 °C for 5 s and combined annealing/extension 60 °C for 10 s. Amplification was followed by a melting curve analysis with continual fluorescence data acquisition during the 60–95 °C melt. A no template control was included in each experiment. Expression values were normalized to the endogenous actin control by calculating the ΔCt (ΔCt = Ct target gene − Ct endogenous control). Data are presented as fold changes, using 2^−ΔCt^ [61–63]. Changes of mango genes expression were analysed by MapMan software (http://mapman.gabipd.org, version 3.5.1) [64].

### Statistical analysis

Statistical analysis was done with GraphPad PRISM 5 (GraphPad Software, San Diego, USA). Significance was determined using one-way analysis of variance with the Tukey’s test for all pairwise multiple comparisons for normally distributed data of equal variance. Standard deviations of ΔCt values were calculated from measurements performed in triplicate.

## Additional file

Additional file 1:
**List of primers used for quantitative reverse transcription-polymerase chain reaction.** (DOCX 32 kb)

## Abbreviations

4CL: 4-coumarate:CoA ligase
ANR: Anthocyanidin reductase
ANS: Anthocyanidin synthase
C3’H: *p*-coumaroyl ester 3′-hydroxylase
C4H: Cinnamate 4-mono-oxygenase
CHI: Chalcone isomerase
CHS: Chalcone synthases
DFR: Dihydroflavonol 4-reductases
EST: Expressed sequence tag
F3H: Flavanone 3-hydroxylase
F3’H: Flavonoid 3’-hydroxylase
FGS: Flavan-3-ol-gallate synthase
FLS: Flavone synthase
GAE: Gallic acid equivalents
KP: Kensington Pride
IW: Irwin
NDM: Nam Doc Mai
PA: Proanthocyanidin
PAL: Phenylalanine ammonia lyase
PF: Phenylpropanoid-flavonoid
SNP: Single nucleotide polymorphisms
