# Supplementary material for: Sequence diversity and differential expression of major phenylpropanoid-flavonoid biosynthetic genes among three mango varieties
Source: BMC Genomics. 2015 Jul 30;16(1):561. doi: 10.1186/s12864-015-1784-x (PMC4518526; doi:10.1186/s12864-015-1784-x)
Supplement: Additional file 1: — List of primers used for quantitative reverse transcription-polymerase chain reaction. (DOCX 32 kb) [file 12864_2015_1784_MOESM1_ESM.docx]

Additional file 1 List of primers used for quantitative reverse transcription-polymerase chain reaction

| **Primer** | **Sequences** |
| --- | --- |
| PAL-F | 5′-TGGATTCAAGGGTGCTGAAATCGC-3′ |
| PAL-R | 5′-TCACATCTTGGTTGTGTTGCTCGG-3′ |
| C4H-F | 5′-TCCCTTTGGAGTTGGCAGAAGAAGC-3′ |
| C4H-R | 5′-TGTCCTGGTGGAGGCAGCA-3′ |
| 4CL-F | 5′-AGATGAGAACGATTTGCCGGAGGT-3′ |
| 4CL-R | 5′-GCTTGTAACGAGTCCTTTGTGCGT-3′ |
| C3'H-F | 5′-AGGAGCTTGACCGAGTGATTGGAT-3′ |
| C3'H-R | 5′-TGAGGCAGCATTAGTGGAGTTGGA-3′ |
| CHS1-F | 5′-AGCTCTTGGGCCTTCGTCCA-3′ |
| CHS1-R | 5′-CGAGCACCTTTGTTGTTCTCAGCC-3′ |
| CHS2-F | 5′-ATCGGCATCAACGACTGGAACTCA-3′ |
| CHS2-R | 5′-GAACTTGGCGGGTTGCTCTCATTT-3′ |
| F3H-F | 5′-GCAAGAAGGGCGGCTTCATAGT-3′ |
| F3H-R | 5′-TTGTCCGGCCACCGTGAATAATCT-3′ |
| F3'H-F | 5′-CGGTGACTTTATCCCGTCCCTCG-3′ |
| F3'H-R | 5′-TCCGTCTGTTTTCCCTTGGCATCA-3′ |
| DFR-F | 5′-CCCACAAAGTTCAAAGGCGTGGAT-3′ |
| DFR-R | 5′-GCTCTGCAAGTCTCCACAGCTCC-3′ |
| ANS-F | 5′-ACTGGCTCTTGGTGTGGAGGC-3′ |
| ANS-R | 5′-TGGAACACACTTTGCAGTGACCCA-3′ |
| ANR-F | 5′-TGGAACAGGTTTGGTGATGGACGA-3′ |
| ANR-R | 5′-TTCCAAGCATGCCTTTCAGCCAAG-3′ |
| Actin-F | 5′-GCTTGCCTATGTTGCCCTTGACTA-3′ |
| Actin-R | 5′-GCATCGGAATCTCTCAGCTCCAAT-3′ |
